# IL-1R1 Blockade Enhances CD40 Agonist-Mediated Immune Responses but Fails to Increase Efficacy or Mitigate Hepatotoxicity in Pancreatic Cancer

**DOI:** 10.1101/2025.02.23.639774

**Published:** 2025-02-27

**Authors:** Akash Boda, Irfan N Bandey, Saikat Chowdhury, Sadhna Aggarwal, Mekala Venugopala, Natalie Wall Fowlkes, Jason Roszik, Michael A Curran, Van Karlyle Morris, Scott Kopetz, Manisha Singh

**Affiliations:** 1 Department of Immunology, The university of Texas MD Anderson Cancer Center, Houston, Texas, USA; 2 Department of Gastrointestinal Medical Oncology, The university of Texas MD Anderson Cancer Center, Houston, Texas, USA; 3 Department of Thoracic Radiation Oncology, The university of Texas MD Anderson Cancer Center, Houston, Texas, USA; 4 Institute for Clinical and Translational Research, Dan L Duncan Comprehensive Cancer Center, Baylor College of Medicine, Houston, Texas, USA; 5 Department of Veterinary Medicine and Surgery, The university of Texas MD Anderson Cancer Center, Houston, Texas, USA; 6 Department of Melanoma, The university of Texas MD Anderson Cancer Center, Houston, Texas, USA

## Abstract

Pancreatic ductal adenocarcinoma (PDAC) has a dismal 5-year survival rate of only 10% in the United States. While immune checkpoint blockade (ICB) has shown efficacy in many solid tumors, PDAC remains largely unresponsive. Agonistic CD40 antibodies can activate PDAC-associated myeloid cells, enhancing innate and adaptive anti-tumor immunity. However, clinical trials with agonistic CD40 antibodies have demonstrated only modest efficacy and significant hepatotoxicity. We previously reported that IL-1 pathway blockade enhances CD40 agonist efficacy against melanoma by depleting polymorphonuclear myeloid-derived suppressor cells (PMN-MDSCs; CD11b^+^Ly6C^+^Ly6G^+^). Since PMN-MDSCs also cause liver toxicity. This study investigates the impact of IL-1R1 blockade on the efficacy and toxicity of CD40 agonist therapy in PDAC.

We found that treatment with an agonistic CD40 antibody induced immune activation and significantly prolonged survival in orthotopic PDAC-bearing mice. The transcriptomic profile revealed that IL-1R1 blockade as monotherapy downregulated innate and adaptive immune response and exacerbated tumor growth. However, the combination therapy of agonistic CD40 antibody and IL-1R1 blockade upregulated several immune-related pathways (GO enrichment analysis), including JAK-STAT signaling, antigen processing, and presentation, natural killer cell mediated cytotoxicity, boosted innate and adaptive immune responses, compared to CD40 agonist treatment alone. Paradoxically, despite these immunostimulatory effects, IL-1R1 blockade failed to improve the overall anti-tumor efficacy of CD40 agonist therapy and exacerbated liver toxicity. Liver toxicity was assessed by serum ALT and AST levels, and transcriptomic profiling of the liver revealed several pathways related to immune infiltration, liver metabolism, viral carcinogenesis and alcoholic liver disease were associated with hepatotoxicity.

To further investigate the role of PMN-MDSCs in PDAC and understand the failure of anti-IL-1R1 therapy, we depleted these cells using an anti-Ly6G antibody in mice treated with an agonistic CD40 antibody. Unexpectedly, Ly6G depletion reduced the efficacy of CD40 agonist therapy, suggesting that, contrary to expectations, Ly6G^+^ immune cells (PMN-MDSCs or neutrophils) exhibit an anti-tumor rather than an immunosuppressive role in PDAC.

Our findings underscore the complex role of IL-1 signaling in modulating immune responses in PDAC and caution against pursuing IL-1R1 blockade, either as a monotherapy or in combination with CD40 agonist antibodies, in future clinical trials for PDAC.

## Introduction:

Pancreatic ductal adenocarcinoma (PDAC) is incurable and has a 5-year survival rate of only 10% in the United States ^1^. PDAC is predicted to become the second leading cause of cancer-related death by 2030 ^2^. The primary cause of the high lethality of PDAC is resistance to therapy. Immunotherapy, such as immune checkpoint blockade (ICB), like anti-PD-1 antibody, is effective against many solid tumors, but PDAC does not respond to ICB ^3, 4^.

PDAC is characterized by extensive accumulation of myeloid cells. Agonistic CD40 antibody therapy activates PDAC-associated myeloid cells through immune co-stimulatory receptor CD40, inducing both innate and tumor-specific immunity and sensitizing the tumor microenvironment (TME) to other immunotherapies^5, 6^. Therefore, the agonistic CD40 antibody is considered the most promising immunotherapy for PDAC. However, clinical trials of several CD40 agonists with or without ICB showed only moderate therapeutic efficacy, and most patients had relapse after an initial response ^7–9^. The combination of CD40 agonist and ICB induces dose-limiting immune-related adverse events (irAEs), such as cytokine storm and liver damage ^10–13^. To enhance the anti-tumor activity and lower the toxicity of the CD40 agonist, it is crucial to discover and target the molecules and pathways that cause resistance to such therapy and therapy-mediated toxicity.

Inflammation is associated with cancer initiation and progression ^14^; however, the underlying mechanism is not clear. In addition, the role of inflammation in resistance to immunotherapies has not been well studied. Interleukin-1 (IL-1) is a major proinflammatory cytokine present in the inflammatory TME ^15^. It increases tumor invasiveness and metastasis. The 2 forms of IL-1, IL-1α, and IL-1β, are derived from different genes but are functionally similar, and both bind to IL-1 receptor type 1 (IL-1R1) ^16, 17^. IL-1 induces accumulation of myeloid-derived suppressor cells (MDSCs) ^18^ and regulatory T cells (Tregs) ^19^ at the site of inflammation, but the importance of this mechanism in the TME is not well studied. IL-1 induces the production of IL-6, IL-8, IL-17, and CRP ^20–22^, which are associated with irAEs and are also associated with the acquisition of resistance to immunotherapies ^23–27^.

We previously reported that CD40 agonist induced IL-1α in the melanoma microenvironment, which conferred adaptive resistance to CD40 agonist therapy through polymorphonuclear MDSCs (PMN-MDSCs) ^28^. Although phenotypically similar to activated neutrophils, PMN-MDSCs are pathologically activated and exhibit immunosuppressive properties. In the tumor microenvironment (TME), activated neutrophils contribute to antitumor immunity, whereas immunosuppressive PMN-MDSCs promote tumor growth^29^.

Agonistic CD40 antibody induces liver damage through tumor-derived inflammatory MDSCs and PMN-MDSCs ^12, 30^. We reported that inflammatory monocytes were the key cells that produced IL-1 in response to CD40 agonist and IL-1-derived PMN-MDSCs in the tumor ^28^.

This study will determine the effect of IL-1R1 blockade on the efficacy and toxicity of agonistic CD40 antibody antibody therapy in PDAC.

## Results:

### Agonistic CD40 antibody treatment reshapes the tumor microenvironment and improves survival in an orthotopic PDAC mouse model

**Figure 1.**
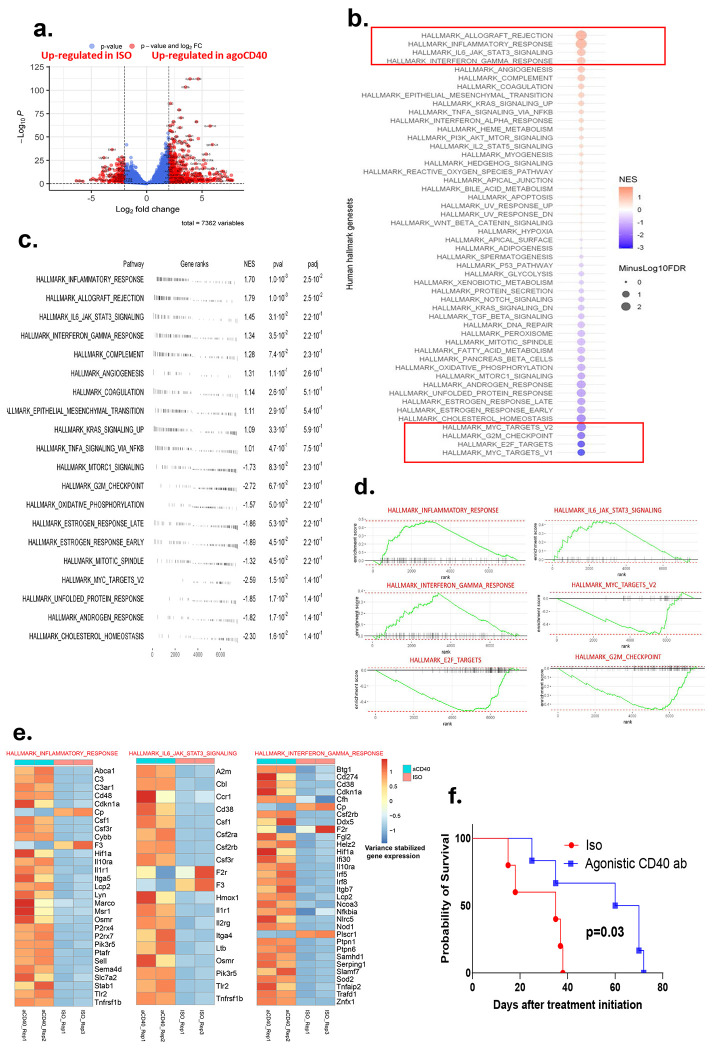
Agonistic CD40 Antibody Induces Immune Activation and Prolongs Survival in PDAC-Bearing Mice. Mice bearing 9-day-old orthotopic PDAC tumors (confirmed by luminescence imaging) were treated every 3 days with agonistic CD40 antibody or isotype control (200 μg/mouse, intraperitoneal). Bulk transcriptomic analysis of tumors was performed after 2 weeks of treatment initiation (n = 2 mice/group). (a) Volcano plot highlighting differentially expressed genes (DEGs) between CD40-treated and isotype control tumors. (b) Gene Set Enrichment Analysis (GSEA) of bulk RNA-seq data showing hallmark pathways enriched in CD40-treated tumors compared to controls. Upregulated pathways included inflammatory response, IFNγ response, and IL-6/JAK-STAT signaling, while oncogenic pathways such as MYC targets V2, E2F targets, and G2M checkpoint were significantly downregulated (FDR < 0.05). (c) Summary of gene ranks, normalized enrichment scores (NES), p-values, and adjusted p-values (padj) for hallmark pathways. (d) Enrichment plots for select hallmark pathways, illustrating the distribution of pathway-associated genes in the ranked gene list. (e) Heatmap of hallmark gene signatures, depicting relative expression across samples, normalized by row Z-scores. (f) Kaplan-Meier survival curves of orthotopic PDAC-bearing mice treated with CD40 antibody or isotype control (log-rank test, n = 5-6 mice/group).

To assess the therapeutic potential of CD40 agonist treatment in pancreatic ductal adenocarcinoma (PDAC), we first evaluated its activity and efficacy in an orthotopic PDAC mouse model. Bulk transcriptomic analysis revealed a significantly higher number of differentially expressed genes in the CD40 agonist-treated group compared to the isotype control group, with 7,362 genes showing altered expression (Fig. 1a).

Pathway enrichment analysis highlighted a profound immunomodulatory effect of CD40 agonist treatment. Key immunologic hallmark pathways, including the inflammatory response, IFNγ response, and IL-6/JAK-STAT signaling pathways, were significantly upregulated (p < 0.01), suggesting a robust activation of both innate and adaptive immunity. In contrast, oncogenic pathways such as MYC targets V2, E2F targets, and the G2M checkpoint were markedly downregulated (p < 0.01), indicating potential tumor-suppressive effects at the transcriptional level (Fig. 1b–c). Gene Set Enrichment Analysis (GSEA) further confirmed these findings (Fig. 1d), and genes associated with these immune-related pathways are detailed in Fig. 1e.

Importantly, CD40 agonist treatment led to a significant extension in survival of orthotopic PDAC-bearing mice compared to isotype antibody-treated controls (Fig. 1f). This suggests that CD40 agonist therapy not only remodels the tumor microenvironment by promoting pro-inflammatory and anti-tumor immune responses but also effectively suppresses oncogenic signaling, ultimately inhibiting tumor progression and improving survival outcomes.

### IL-1R1 blockade enhances agonistic CD40 antibody induced immunity but failed to improve anti-tumor efficacy in PDAC

**Figure 2.**
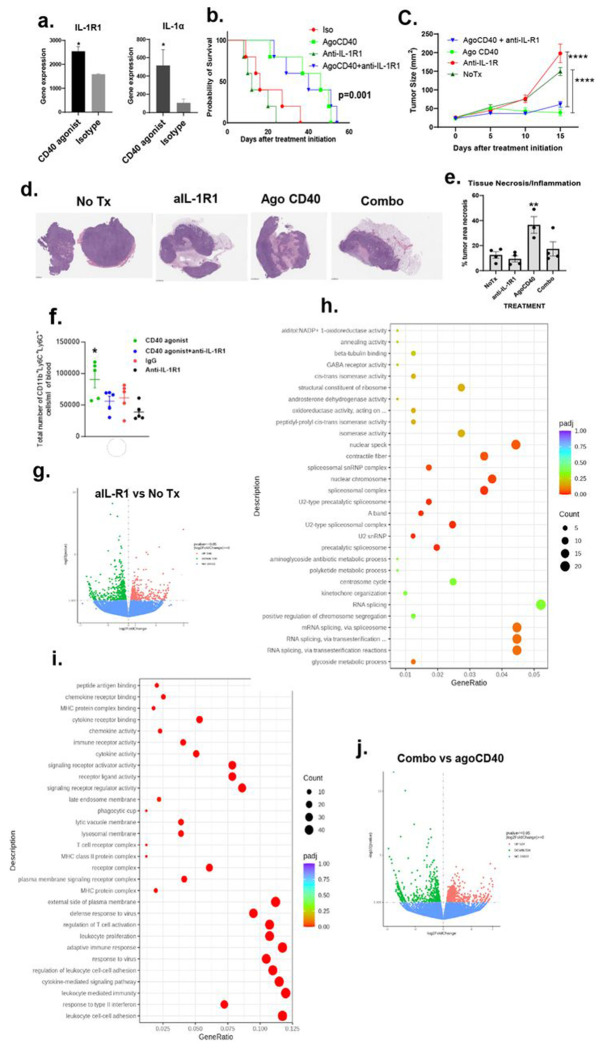

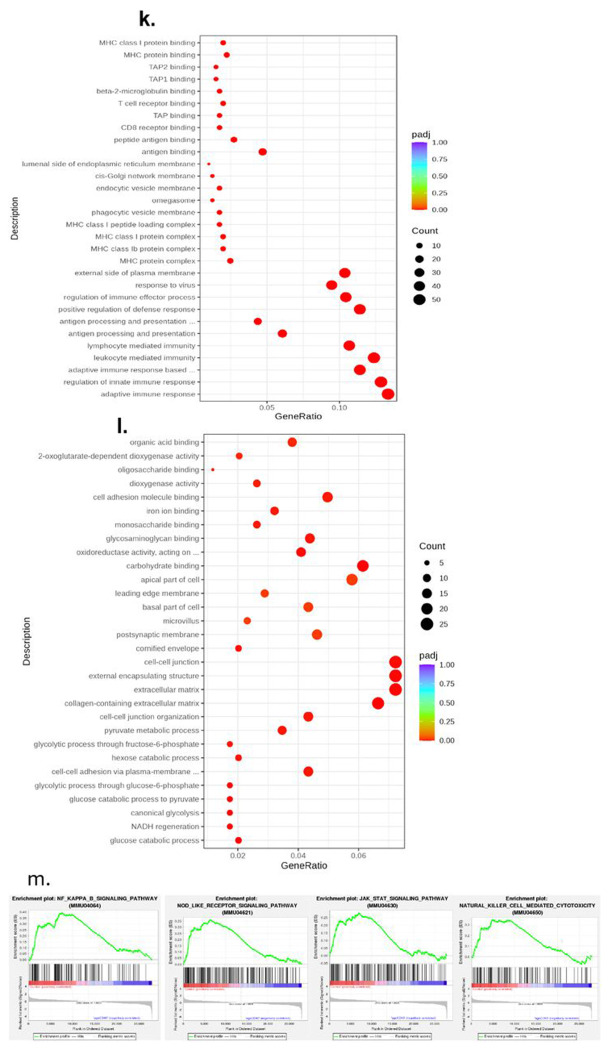
Combination Therapy with CD40 Agonist and Anti-IL-1R1 Antibodies Enhances Immune Activation but Fails to Improve Therapeutic Efficacy in PDAC Compared to CD40 Monotherapy. Mice bearing 9-day-old orthotopic PDAC tumors (confirmed by luminescence imaging) were treated every 3 days with the indicated antibodies (200 μg/mouse, intraperitoneal). (a) Bulk transcriptomic analysis of orthotopic PDAC tumors after 2 weeks of treatment initiation (*p < 0.05; unpaired t-test, n = 2 mice/group). (b) Kaplan-Meier survival curves (log-rank test, n = 5-6 mice/group). (c) Tumor growth kinetics in subcutaneous PDAC-bearing mice treated every 3 days with agonistic CD40 antibody, anti-IL-1R1 antibody, their combination, or left untreated (****p < 0.0001; two-way ANOVA, n = 5-8 mice/group). (d) Representative H&E-stained tumor sections showing necrosis after 2 weeks of treatment (n = 3-4 mice/group). (e) Quantification of necrotic areas (**p < 0.01; one-way ANOVA, n = 3-4 mice/group). (f) Flow cytometric analysis of PMN-MDSCs in peripheral blood after 2 weeks of treatment (*p < 0.05; unpaired Student’s t-test, n = 5 mice/group). (g) Volcano plot of differentially expressed genes (DEGs) in subcutaneous PDAC tumors treated with anti-IL-1R1 versus untreated controls (red: upregulated; green: downregulated; 546 up, 530 down; p ≤ 0.05, log2FC ≥ 0). (h–i) Gene Ontology (GO) enrichment analysis of DEGs from (g), showing significantly upregulated (h) and downregulated (i) biological processes. (j) Volcano plot of DEGs comparing combination therapy (CD40 + anti-IL-1R1) versus CD40 monotherapy (524 up, 534 down; p ≤ 0.05, log2FC ≥ 0). (k–l) GO enrichment analysis of DEGs from (j), highlighting upregulated (k) and downregulated (l) pathways. (m) Gene Set Enrichment Analysis (GSEA) plots demonstrating the enrichment of indicated gene sets in the combination therapy group compared to CD40 monotherapy.

To assess the impact of IL-1R1 blockade on agonistic CD40 antibody efficacy, we first examined the expression of IL-1R1, and IL-1α/β in orthotopic PDAC tumors. Notably, these tumors expressed IL-1R1 and IL-1α in response to agonistic CD40 antibody (Fig. 2a). Next, we evaluated the anti-tumor efficacy of anti-IL-1R1 antibody, either as monotherapy or in combination with an agonistic CD40 antibody, in both orthotopic and subcutaneous PDAC models. We found that anti-IL-1R1 monotherapy reduced survival in orthotopic PDAC-bearing mice compared to the isotype control, while the combination of anti-IL-1R1 and agonistic CD40 antibodies therapy did not improve outcomes over CD40 agonist treatment alone (Fig. 2b). Similarly, in subcutaneous PDAC models, anti-IL-1R1 treatment accelerated tumor growth compared to the untreated group, and the combination of anti-IL-1R1 and CD40 agonist therapy performed worse than CD40 agonist monotherapy (Fig. 2c). Histological analysis revealed significant more necrosis in the agonistic CD40 antibody treated group than in the combination therapy group ((Fig. 2d–e).

Previously, we reported that IL-1R1 blockade reduces the number of PMN-MDSCs in melanoma ^28^. To determine whether this effect extends to PDAC, we analyzed PMN-MDSC levels in PDAC-bearing mice. Indeed, treatment with an anti-IL-1R1 antibody significantly reduced the number of PMN-MDSCs in the blood of CD40 agonist-treated PDAC-bearing mice (Fig. 2f).

Next, we examined the impact of IL-1R1 blockade on the tumor microenvironment by comparing the bulk transcriptomes of PDAC tumors. Specifically, we analyzed tumors treated with an anti-IL-1R1 antibody versus untreated controls, as well as tumors receiving a combination of agonistic CD40 and anti-IL-1R1 antibody versus CD40 agonist monotherapy. Through Gene Ontology (GO) enrichment analysis of differentially expressed genes (p ≤ 0.05) (Fig. 2g), The anti-IL-1R1 antibody group showed upregulation of genes related to the spliceosome, spliceosome complex, and mRNA splicing compared to the No Tx group (padj = 0.00-0.1) (Fig. 2h). In contrast, we identified several immune-related pathways and molecules were significantly downregulated in the anti-IL-1R1 group compared to the no-treatment (No Tx) group. These included response to type II interferon, leukocyte-mediated immunity, cytokine-mediated signaling pathways, adaptive immune response, and regulation of T cell activation (padj = 0.00) (Fig. 2i).

When comparing combination therapy (agonistic CD40 plus anti-IL-1R1 antibodies) to agonistic CD40 monotherapy using Gene Ontology (GO) enrichment analysis of differentially expressed genes (p ≤ 0.05) (Fig. 2j), we found that the combination therapy group was enriched for activated innate and adaptive immune responses. This included processes such as antigen processing and presentation, leukocyte-mediated immunity, lymphocyte-mediated immunity, adaptive immune responses, and regulation of immune effector processes (padj = 0.00) (Fig. 2k). In contrast, many metabolic pathways were downregulated in the combination therapy group (padj = 0.00) (Fig. 2l).

Furthermore, Gene Set Enrichment Analysis (GSEA) confirmed the enrichment of genes involved in NOD-like receptor signaling, nuclear factor kappa B (NF-κB) signaling, natural killer cell-mediated cytotoxicity, and the JAK-STAT signaling pathway in the combination therapy group compared to the agonistic CD40 monotherapy group (Fig. 2m).

Taken together, these findings suggest that while combination therapy with agonistic CD40 and anti-IL-1R1 antibodies elicits a more robust immune response than agonistic CD40 monotherapy, it does not enhance the anti-tumor efficacy of the agonistic CD40 antibody.

### IL-1R1 blockade exacerbates CD40 agonist induces hepatotoxicity:

Agonistic CD40 antibody induces liver damage through tumor-derived PMN-MDSCs^12, 30^. We found that blocking the IL-1 pathway reduces the number of CD40 agonist-induced polymorphonuclear myeloid-derived suppressor cells (PMN-MDSCs) in the blood (Fig. 2e). Based on this observation, we hypothesized that inhibiting the IL-1 pathway using an anti-IL-1R1 antibody might mitigate CD40 agonist-induced hepatotoxicity.

To investigate whether CD40 agonist-induced hepatotoxicity is mediated by IL-1, we measured serum levels of aspartate aminotransferase (AST) and alanine aminotransferase (ALT) markers of liver injury in PDAC-bearing mice treated with either CD40 agonist monotherapy or CD40 agonist in combination with anti-IL-1R1 antibody. Measurements were taken 48 hours, 9 days, and 3 weeks after treatment initiation.

**Figure 3.**
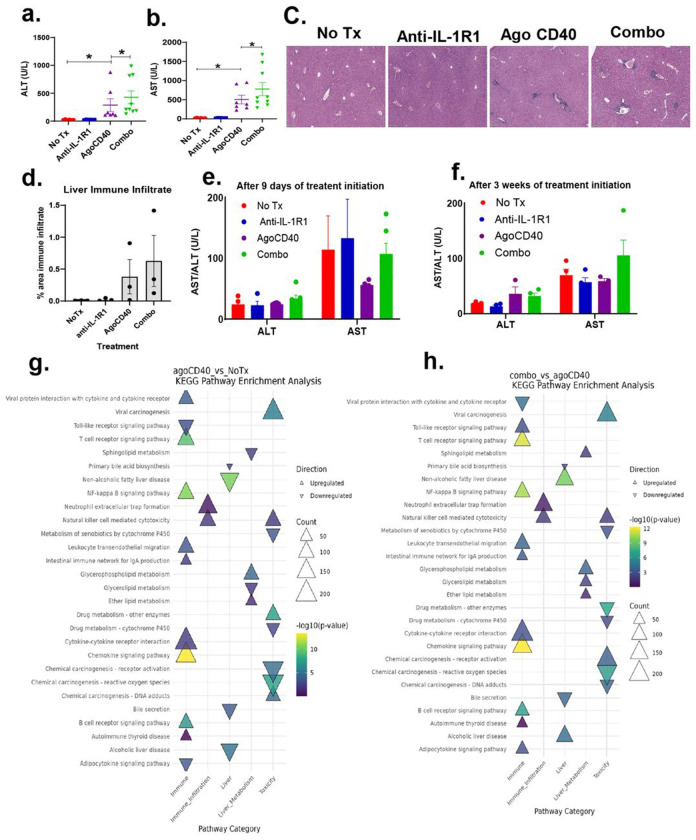
Anti-IL-1R1 blockade exacerbates agonistic CD40 antibody-induced hepatoxicity. Mice bearing 9-day-old subcutaneous PDAC tumors were treated as indicated. (a-b) Serum ALT (a) and AST (b) levels were measured 48 hours after treatment initiation using the COBAS INTEGRA 400 Plus analyzer. *p < 0.01, Wilcoxon non-parametric test; n = 6-9 mice/group. Data represent results combined from two independent experiments. (c) Representative H&E-stained liver histology images from mice collected one week after treatment initiation (n = 3 mice/group, two treatments administered). (d) Cumulative quantification of immune infiltration and histological changes (n = 3 mice/group). (e-f) Serum ALT and AST levels measured after 9 days (e) and 3 weeks (f) of treatment initiation. Combo: agonistic CD40 + anti-IL-1R1 antibody. (g-h) Transcriptomic profiling of the liver from PDAC-bearing mice one week after treatment initiation (two treatments total). KEGG pathway enrichment analysis comparing agonistic CD40 vs. no treatment (g) and combo vs. agonistic CD40 (h). The dot plot highlights pathways associated with liver function, toxicity, and immune infiltration. Dot size represents the number of genes involved (Count), while color intensity reflects statistical significance (-log10 p-value). Upregulated pathways are marked with upward triangles (▲), and downregulated pathways with downward triangles (▼). Pathways with p-value < 0.05 are considered significantly enriched.

At 48 hours, both AST and ALT levels were significantly elevated in CD40 agonist-treated mice compared to untreated controls. Unexpectedly, the addition of anti-IL-1R1 antibody to CD40 agonist therapy further exacerbated AST and ALT elevations compared to CD40 agonist alone (Fig. 3a–b). Histological analysis (H&E staining) of liver sections at 48 hours further supported this finding, revealing increased immune infiltration in both CD40 agonist-treated mice (vs. untreated controls) and combination therapy-treated mice (vs. CD40 monotherapy) (Fig. 3c–d). These results indicate that IL-1 pathway blockade worsens CD40 agonist-induced hepatotoxicity.

Interestingly, after 9 days and 3 weeks of treatment (with dosing every 3 days), AST and ALT levels were no longer elevated in either the monotherapy or combination therapy groups (Fig. 3e–f). This suggests that CD40 agonist-induced hepatotoxicity is transient and resolves over time.

To investigate the mechanisms underlying hepatotoxicity induced by agonistic CD40 antibody monotherapy and its combination with anti-IL-1R1 therapy, we performed transcriptomic profiling of liver tissues. To interpret the biological significance of differentially expressed genes, we conducted KEGG pathway enrichment analysis.

Our analysis identified genes and pathways associated with liver function, toxicity, and immune infiltration in both monotherapy and combination therapy groups. When comparing agonistic CD40 antibody treatment to the untreated control, we observed significant upregulation of pathways related to B cell receptor signaling, chemokine receptor signaling, natural killer (NK) cell-mediated cytotoxicity, neutrophil extracellular trap formation, NF-κB signaling, glycerophospholipid metabolism, and viral carcinogenesis (Fig. 3g).

In contrast, the comparison between combination therapy (CD40 agonist + anti-IL-1R1) and CD40 agonist monotherapy revealed enrichment of similar immune-related pathways, including B cell receptor signaling, chemokine receptor signaling, NK cell-mediated cytotoxicity, neutrophil extracellular trap formation, NF-κB signaling, and viral carcinogenesis. However, unlike the monotherapy group, the combination therapy group also exhibited significant upregulation of pathways related to alcoholic liver disease, non-alcoholic fatty liver disease (NAFLD), and chemical carcinogenesis-receptor activation (Fig. 3h), suggesting an increased risk of liver toxicity with combination treatment.

### Ly6G^+^ Cells (PMN-MDSCs/Neutrophils) Exhibit Anti-Tumor Effects in PDAC:

While agonistic CD40 antibody enhances anti-tumor immunity, it also induces the expansion of polymorphonuclear myeloid-derived suppressor cells (PMN-MDSCs), which are typically associated with immunosuppressive effects on anti-tumor responses. To counteract this, we investigated the impact of anti-IL-1R1 therapy, which effectively reduced CD40 agonist-induced PMN-MDSCs (CD11b^+^Ly6C^+^Ly6G^+^) (Fig. 2e). However, despite this reduction, anti-IL-1R1 therapy failed to enhance the efficacy of the CD40 agonist (Fig. 2b & 2c).

**Figure 4.**
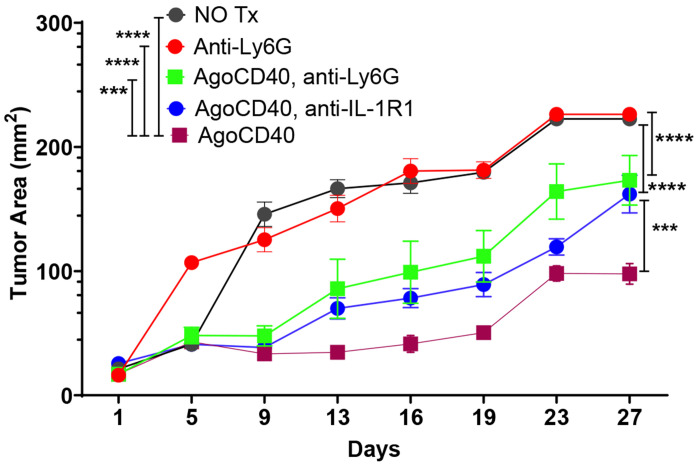
Ly6G^+^ Cells (PMN-MDSCs/Neutrophils) Exhibit Anti-Tumor Effects in PDAC. Mice bearing 9-day-old subcutaneous PDAC tumors were treated as indicated. Graphs show tumor growth at indicated time points. Figure: Tumor growth kinetics in mice bearing 9-day-old subcutaneous PDAC tumors treated as indicated. Tumor area (mm^2^) was measured over 27 days following treatment initiation. Treatment groups included: no treatment (NO Tx), anti-Ly6G, agonistic CD40 (AgoCD40), AgoCD40 combined with anti-Ly6G, and AgoCD40 combined with anti-IL-1R1. Data represent mean ± SEM (n = 6-9 mice per group). Statistical significance was determined by two-way ANOVA with Tukey’s post hoc test. ***p < 0.001, ****p < 0.0001.

To further clarify whether Ly6G^+^ cells play a pro-tumorigenic or anti-tumorigenic role in PDAC, we selectively depleted Ly6G^+^ cells and assessed their impact on tumor growth. Unexpectedly, Ly6G^+^ cell depletion accelerated tumor progression (Fig. 4).

These findings challenge the traditional view of Ly6G^+^ cells as purely immunosuppressive and suggest that, in the context of PDAC, Ly6G^+^ cells may have a protective, anti-tumor immune function.

### IL-1R1, IL-1α, and IL-1β expression and association with survival in Human PDAC:

**Figure 5.**
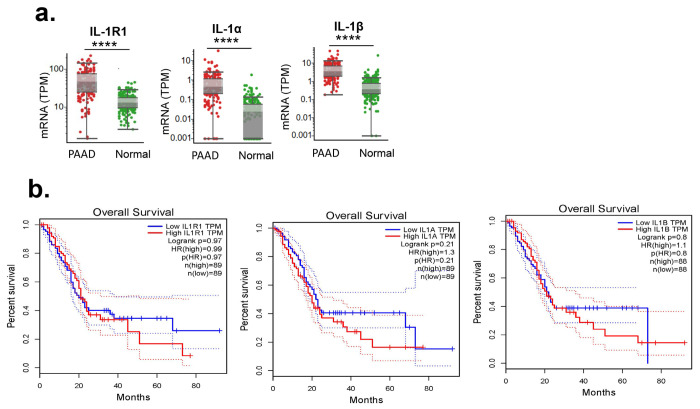
Expression of IL1 family genes and their association with Overall Survival in Pancreatic adenocarcinoma (PAAD) Patients. (a) Box plots showing the expression levels of IL1R1, IL1α, andIL1β in patient samples. Gene expression (TPM: transcripts per million) from RNA-Seq in TCGA-PAAD samples and normal pancreas****p < 0.0001; Student’s t-test Each dot represents an individual sample. The median expression is indicated by the horizontal line within each box, with whiskers representing the interquartile range. (B) Kaplan-Meier survival curves depicting overall survival based on high (red line) and low (blue line) expression of IL1R1, IL1α, and IL1β. Dotted lines indicate 95% confidence intervals. Log-rank p-values and hazard ratios (HR) for each gene are provided in the figure. IL1R1: No significant difference in overall survival (log-rank p = 0.97, HR = 0.99). IL1α: No significant difference in overall survival (log-rank p = 0.21, HR = 1.3). IL1β: No significant difference in overall survival (log-rank p = 0.8, HR = 1.1). Each survival analysis was performed with nearly equal sample sizes between high and low expression groups (n ≈ 88–89 per group).

To enhance the translational relevance of our preclinical findings, we examined the expression of IL-1R1, IL-1α, and IL-1β in human pancreatic ductal adenocarcinoma (PDAC). Our analysis revealed that their expression levels were significantly higher in PDAC tumors compared to normal pancreatic tissue (Fig. 5a), suggesting a potential role for IL-1 signaling in pancreatic cancer.

However, survival analysis indicated that IL-1R1, IL-1α, and IL-1β expression levels did not significantly impact overall Survival in PDAC patients (Fig. 5b). These findings suggest that targeting the IL-1 pathway may have limited therapeutic benefit for PDAC patients.

## Discussion:

Pancreatic ductal adenocarcinoma (PDAC) remains one of the most lethal malignancies, largely due to its resistance to conventional therapies, including immune checkpoint blockade (ICB) with anti-PD-1 antibodies. Agonistic CD40 antibodies have emerged as promising immunotherapeutic agents by activating both innate and adaptive anti-tumor immunity. While CD40 agonists have demonstrated efficacy across several solid tumors, including PDAC, clinical responses remain modest, with only a few cases achieving durable outcomes when administered alone or in combination with ICB.

Polymorphonuclear myeloid-derived suppressor cells (PMN-MDSCs; CD11b^+^Ly6G^+^) are well-established contributors to the immunosuppressive tumor microenvironment in PDAC. Beyond dampening anti-tumor immunity, PMN-MDSCs have also been implicated in treatment-associated liver toxicity. Notably, no clinically approved therapeutics specifically target PMN-MDSCs in humans, underscoring an unmet need for strategies to mitigate their immunosuppressive effects without compromising host defenses.

Previously, we demonstrated that CD40 agonist therapy induces interleukin-1α (IL-1α) within the tumor microenvironment, promoting adaptive resistance through the expansion of PMN-MDSCs. Moreover, in melanoma models, IL-1R1 blockade enhanced CD40 agonist efficacy by depleting PMN-MDSCs, suggesting a critical role for IL-1 signaling in driving immune suppression. Based on these findings, we hypothesized that IL-1R1 blockade would potentiate CD40 agonist efficacy while mitigating liver toxicity in PDAC by reducing PMN-MDSC infiltration.

Our study is the first to determine the effect of IL-1R1 blockade on the efficacy and toxicity of agonistic CD40 antibody therapy in PDAC.

However, our current study revealed striking tumor-specific differences. In contrast to melanoma, IL-1R1 blockade failed to enhance CD40 agonist efficacy in orthotopic PDAC models. Surprisingly, CD40 monotherapy outperformed combination therapy in the subcutaneous PDAC model, while IL-1R1 blockade alone significantly worsened survival compared to isotype control. These findings highlight fundamental context-dependent roles of IL-1 signaling, suggesting that its impact on tumor immunity is tumor-type specific and influenced by the local microenvironment.

Further investigations into the role of Ly6G^+^ cells (PMN-MDSCs/neutrophils) provided additional insights. Selective depletion of Ly6G^+^ cells unexpectedly compromised CD40 agonist efficacy, accelerating tumor growth and reducing survival in PDAC-bearing mice. This finding challenges the traditional view of PMN-MDSCs as purely pro-tumorigenic, suggesting that Ly6G^+^ cells may possess anti-tumorigenic properties within the PDAC microenvironment.

Our findings align with those of Fan et al.^31^, who demonstrated that tumor-derived interleukin-1 receptor antagonist (IL-1Ra) exhibits immunosuppressive functions and promotes pancreatic cancer progression. This further underscores the complex role of IL-1 signaling in PDAC and its impact on immune modulation and tumor growth.

Recent reports showed that tumor cell-derived IL-1β induces immune suppression in the pancreatic cancer microenvironment. Das et al.^32^ reported that neutralizing IL-1β enhanced the efficacy of anti-PD-1, and Takahashi et al.^33^ reported that IL-1β promoted tumorigenesis through immune-suppressive B cells. Aggen et al. recently showed that the combination of IL-1β blockade and either anti-PD-1 or a tyrosine kinase inhibitor had greater anti-tumor activity than monotherapy alone against mouse renal cell carcinoma ^34^. We cannot directly correlate our findings with the findings as mentioned above because IL-1R1 blockade inhibits both IL-1α and IL-1β pathways, not only IL-1β.

Our preclinical findings support Herremans et al ^35^. report who used The Cancer Genome Atlas (TCGA) and Gene Expression Omnibus databases to study IL-1 in PDAC and found that IL-1 was associated with significant changes in the tumor immune landscape and the expression of tumor immune checkpoint proteins.

Dosch et al.^36^ found that the human IL-1R1 antagonist anakinra significantly reduced IL-6 and activated STAT3 levels in pancreatic tumors from PKT mice, and combining anakinra with chemotherapy markedly extended overall survival compared to vehicle treatment or anakinra monotherapy in this genetically engineered mouse model (GEMM) of PDAC.

Furthermore, using preclinical tumor models and patient samples, Theivanthiran et al.^37^ found that activation of CD8 T cells through PD-1 blockade induced a PD-L1/NLRP3 inflammasome signaling cascade, which resulted in recruitment of PMN-MDSCs into melanoma and led to suppression of anti-tumor immunity.

IL-1 induces liver inflammation in various liver diseases, like alcoholic and non-alcoholic liver disease, autoimmune hepatitis, and viral hepatitis ^38^. However, the toxic effect of cancer immunotherapy-induced IL-1 on the liver, colon, and other organs has not been studied. Furthermore, although IL-6, IL-8, IL-17, and CRP have been shown to be the major cytokines that cause irAEs in response to cancer immunotherapy and are downstream of IL-1, blockade of the IL-1 pathway to suppress immunotherapy-mediated toxicity has not been explored.

CD40 agonist therapy has been shown to induce liver damage, primarily attributed to tumor-derived PMN-MDSCs^12^, ^30^. Interestingly, our study revealed that while IL-1R1 blockade effectively reduced PMN-MDSC levels in mice, it unexpectedly exacerbated CD40 agonist-induced hepatotoxicity. This finding suggests that CD40-induced liver toxicity is independent of IL-1 signaling or PMN-MDSCs, highlighting a more complex mechanism underlying CD40-mediated hepatotoxicity that warrants further investigation.

Our study underscores the complex and context-dependent nature of IL-1 signaling and PMN-MDSC function in PDAC. While CD40 agonists remain promising, therapeutic strategies aimed at enhancing their efficacy and lowering toxicity must consider the tumor-specific immune landscape to avoid unintended consequences.

## Material and Methods:

### Mice and Tumor Model:

Male C57BL/6 mice (6–8 weeks old) were obtained from Jackson Laboratory and maintained under specific pathogen-free conditions at the institutional animal facility. All experiments were performed in accordance with the guidelines approved by the Institutional Animal Care and Use Committee (IACUC) of MD Anderson Cancer Center.

To establish subcutaneous PDAC tumors, mice were injected subcutaneously into the right flank with 1 × 10^6^ KPC cells (Kras^+/G12D^ TP53^+/R172H^ Pdx1-Cre), a well-established murine PDAC cell line, generously provided by Dr. Michael Curran (MD Anderson Cancer Center). Tumors were allowed to grow for 9 days prior to treatment initiation.

Orthotopic PDAC Tumor Cell Implantation

Orthotopic PDAC tumor implantation was performed using luciferase (Luc)-labeled KPS cells. A total of 30,000 cells were implanted following a standard protocol. Tumor growth was monitored weekly using IVIS bioluminescence imaging, as previously described^39^.

### Treatment Scheme

Tumor presence was confirmed by imaging prior to treatment initiation. Beginning 9 days after tumor implantation, subcutaneous and orthotopic PDAC-bearing mice were treated with anti-IL-1R1 (Clone JAMA-147, BioXCell) or isotype control antibodies (BioXCell) administered intraperitoneally. Twenty-four hours after the initial treatment, the mice received an agonistic CD40 antibody (Clone FGK4.5, BioXCell) or corresponding isotype controls administered intraperitoneally. Treatments were administered every 3 days for 2 weeks, followed by once-weekly dosing. In select experiments, mice received an anti-Ly6G antibody (depletion antibody; Clone 1A8, BioXCell) twice weekly. All antibodies were administered at a dose of 200 μg per injection via the intraperitoneal route.

### Tumor Growth and Survival

Subcutaneous tumor growth was monitored using calipers, with tumor size calculated as the product of perpendicular diameters. Mice were euthanized when tumors reached 200 mm^2^. Orthotopic tumor growth was monitored weekly using IVIS bioluminescence imaging. Mice were sacrificed upon reaching a significant tumor burden. Survival analysis was performed using the log-rank test.

### Serum Analysis

Blood samples were collected via retro-orbital bleeding at three time points: 48 hours after treatment initiation, after 9 days, and after 3 weeks. Serum alanine aminotransferase (ALT) and aspartate aminotransferase (AST) levels were measured using the COBAS INTEGRA 400 Plus analyzer (Roche Diagnostics) to assess liver toxicity.

### Histological Analysis

Liver and tumor tissues were harvested after one and two weeks of treatment. Tissues were fixed in 10% neutral-buffered formalin, embedded in paraffin, and sectioned. Hematoxylin and eosin (H&E) staining was performed to assess histological changes, including tissue architecture, necrosis, and immune cell infiltration.

### Transcriptome Analysis of Liver and Tumor

Bulk RNA sequencing was performed by Novogene (Beijing, China), a leading provider of high-throughput sequencing services. Total RNA was extracted from flash-frozen tissue samples using the RNeasy Mini Kit (Qiagen) according to the manufacturer’s instructions. RNA quality and integrity were assessed using the Agilent 2100 Bioanalyzer, ensuring RNA integrity numbers (RIN) > 7.0 for downstream analysis.

Sequencing libraries were prepared using the NEBNext Ultra II RNA Library Prep Kit (New England Biolabs), incorporating poly(A) enrichment to focus on mRNA transcripts. Libraries were quantified using Qubit and real-time PCR and sequenced on the Illumina NovaSeq 6000 platform, generating 150 bp paired-end reads. Raw data underwent quality control using Fast QC, and clean reads were aligned to the mouse reference genome (GRCm39) using HISAT2. Differential expression analysis was performed with DESeq2, while KEGG and GO pathway enrichment analyses were conducted using the Cluster Profiler R package.

### Statistical Analysis

Data were analyzed using GraphPad Prism 10 software. All results are expressed as mean ± SEM. Tumor growth was evaluated by two-way ANOVA with Tukey’s post hoc test, while non-parametric data were analyzed using the Wilcoxon rank-sum test. Statistical significance was defined as p < 0.05 (p < 0.05; *, p < 0.01; **, p < 0.001; ***, p < 0.0001).

## Data Availability

The datasets generated and analyzed during the current study are available from the corresponding author upon reasonable request.

## References

[1] Mizrahi JD, Surana R, Valle JW, Shroff RT. Pancreatic cancer. Lancet. 2020;395(10242):2008–20. Epub 2020/07/01. doi: 10.1016/S0140-6736(20)30974-0. 32593337

[2] Rahib L, Smith BD, Aizenberg R, Rosenzweig AB, Fleshman JM, Matrisian LM. Projecting cancer incidence and deaths to 2030: the unexpected burden of thyroid, liver, and pancreas cancers in the United States. Cancer Res. 2014;74(11):2913–21. Epub 2014/05/21. doi: 10.1158/0008-5472.CAN-14-0155. 24840647

[3] Kabacaoglu D, Ciecielski KJ, Ruess DA, Algul H. Immune Checkpoint Inhibition for Pancreatic Ductal Adenocarcinoma: Current Limitations and Future Options. Front Immunol. 2018;9:1878. Epub 20180815. doi: 10.3389/fimmu.2018.01878. 30158932 PMC6104627

[4] Schizas D, Charalampakis N, Kole C, Economopoulou P, Koustas E, Gkotsis E, Ziogas D, Psyrri A, Karamouzis MV. Immunotherapy for pancreatic cancer: A 2020 update. Cancer Treat Rev. 2020;86:102016. Epub 20200325. doi: 10.1016/j.ctrv.2020.102016. 32247999

[5] Vonderheide RH. CD40 Agonist Antibodies in Cancer Immunotherapy. Annu Rev Med. 2020;71:47–58. Epub 20190814. doi: 10.1146/annurev-med-062518-045435. 31412220

[6] Beatty GL, Chiorean EG, Fishman MP, Saboury B, Teitelbaum UR, Sun W, Huhn RD, Song W, Li D, Sharp LL, Torigian DA, O’Dwyer PJ, Vonderheide RH. CD40 agonists alter tumor stroma and show efficacy against pancreatic carcinoma in mice and humans. Science. 2011;331(6024):1612–6. doi: 10.1126/science.1198443. 21436454 PMC3406187

[7] Li DK, Wang W. Characteristics and clinical trial results of agonistic anti-CD40 antibodies in the treatment of malignancies. Oncol Lett. 2020;20(5):176. Epub 2020/09/17. doi: 10.3892/ol.2020.12037. 32934743 PMC7471753

[8] Padron LJ, Maurer DM, O’Hara MH, O’Reilly EM, Wolff RA, Wainberg ZA, Ko AH, Fisher G, Rahma O, Lyman JP, Cabanski CR, Yu JX, Pfeiffer SM, Spasic M, Xu J, Gherardini PF, Karakunnel J, Mick R, Alanio C, Byrne KT, Hollmann TJ, Moore JS, Jones DD, Tognetti M, Chen RO, Yang X, Salvador L, Wherry EJ, Dugan U, O’Donnell-Tormey J, Butterfield LH, Hubbard-Lucey VM, Ibrahim R, Fairchild J, Bucktrout S, LaVallee TM, Vonderheide RH. Sotigalimab and/or nivolumab with chemotherapy in first-line metastatic pancreatic cancer: clinical and immunologic analyses from the randomized phase 2 PRINCE trial. Nat Med. 2022;28(6):1167–77. Epub 20220603. doi: 10.1038/s41591-022-01829-9. 35662283 PMC9205784

[9] Weiss SA, Sznol M, Shaheen M, Berciano-Guerrero MA, Couselo EM, Rodriguez-Abreu D, Boni V, Schuchter LM, Gonzalez-Cao M, Arance A, Wei W, Ganti AK, Hauke RJ, Berrocal A, Iannotti NO, Hsu FJ, Kluger HM. A Phase II Trial of the CD40 Agonistic Antibody Sotigalimab (APX005M) in Combination with Nivolumab in Subjects with Metastatic Melanoma with Confirmed Disease Progression on Anti-PD-1 Therapy. Clin Cancer Res. 2024;30(1):74–81. doi: 10.1158/1078-0432.CCR-23-0475. 37535056 PMC10767304

[10] Johnson P, Challis R, Chowdhury F, Gao Y, Harvey M, Geldart T, Kerr P, Chan C, Smith A, Steven N, Edwards C, Ashton-Key M, Hodges E, Tutt A, Ottensmeier C, Glennie M, Williams A. Clinical and biological effects of an agonist anti-CD40 antibody: a Cancer Research UK phase I study. Clin Cancer Res. 2015;21(6):1321–8. Epub 2015/01/16. doi: 10.1158/1078-0432.CCR-14-2355. 25589626

[11] Vonderheide RH, Flaherty KT, Khalil M, Stumacher MS, Bajor DL, Hutnick NA, Sullivan P, Mahany JJ, Gallagher M, Kramer A, Green SJ, O’Dwyer PJ, Running KL, Huhn RD, Antonia SJ. Clinical activity and immune modulation in cancer patients treated with CP-870,893, a novel CD40 agonist monoclonal antibody. J Clin Oncol. 2007;25(7):876–83. Epub 2007/03/01. doi: 10.1200/JCO.2006.08.3311. 17327609

[12] Medina-Echeverz J, Ma C, Duffy AG, Eggert T, Hawk N, Kleiner DE, Korangy F, Greten TF. Systemic Agonistic Anti-CD40 Treatment of Tumor-Bearing Mice Modulates Hepatic Myeloid-Suppressive Cells and Causes Immune-Mediated Liver Damage. Cancer Immunol Res. 2015;3(5):557–66. Epub 20150130. doi: 10.1158/2326-6066.CIR-14-0182. 25637366 PMC4420683

[13] Blake SJ, James J, Ryan FJ, Caparros-Martin J, Eden GL, Tee YC, Salamon JR, Benson SC, Tumes DJ, Sribnaia A, Stevens NE, Finnie JW, Kobayashi H, White DL, Wesselingh SL, O’Gara F, Lynn MA, Lynn DJ. The immunotoxicity, but not anti-tumor efficacy, of anti-CD40 and anti-CD137 immunotherapies is dependent on the gut microbiota. Cell Rep Med. 2021;2(12):100464. Epub 20211208. doi: 10.1016/j.xcrm.2021.100464. 35028606 PMC8714857

[14] Hussain SP, Harris CC. Inflammation and cancer: an ancient link with novel potentials. Int J Cancer. 2007;121(11):2373–80. Epub 2007/09/26. doi: 10.1002/ijc.23173. 17893866

[15] Dinarello CA. Proinflammatory cytokines. Chest. 2000;118(2):503–8. Epub 2000/08/11. doi: 10.1378/chest.118.2.503. 10936147

[16] Voronov E, Shouval DS, Krelin Y, Cagnano E, Benharroch D, Iwakura Y, Dinarello CA, Apte RN. IL-1 is required for tumor invasiveness and angiogenesis. Proc Natl Acad Sci U S A. 2003;100(5):2645–50. Epub 2003/02/25. doi: 10.1073/pnas.0437939100. 12598651 PMC151394

[17] Lewis AM, Varghese S, Xu H, Alexander HR. Interleukin-1 and cancer progression: the emerging role of interleukin-1 receptor antagonist as a novel therapeutic agent in cancer treatment. J Transl Med. 2006;4:48. Epub 2006/11/14. doi: 10.1186/1479-5876-4-48. 17096856 PMC1660548

[18] Lee PY, Kumagai Y, Xu Y, Li Y, Barker T, Liu C, Sobel ES, Takeuchi O, Akira S, Satoh M, Reeves WH. IL-1alpha modulates neutrophil recruitment in chronic inflammation induced by hydrocarbon oil. J Immunol. 2011;186(3):1747–54. Epub 2010/12/31. doi: 10.4049/jimmunol.1001328. 21191074 PMC3607541

[19] Brinster C, Shevach EM. Costimulatory effects of IL-1 on the expansion/differentiation of CD4+CD25+Foxp3+ and CD4+CD25+Foxp3- T cells. J Leukoc Biol. 2008;84(2):480–7. Epub 2008/05/15. doi: 10.1189/jlb.0208085. 18477692 PMC2493074

[20] Cahill CM, Rogers JT. Interleukin (IL) 1beta induction of IL-6 is mediated by a novel phosphatidylinositol 3-kinase-dependent AKT/IkappaB kinase alpha pathway targeting activator protein-1. J Biol Chem. 2008;283(38):25900–12. Epub 2008/06/03. doi: 10.1074/jbc.M707692200. 18515365 PMC2533786

[21] Jung YD, Fan F, McConkey DJ, Jean ME, Liu W, Reinmuth N, Stoeltzing O, Ahmad SA, Parikh AA, Mukaida N, Ellis LM. Role of P38 MAPK, AP-1, and NF-kappaB in interleukin-1beta-induced IL-8 expression in human vascular smooth muscle cells. Cytokine. 2002;18(4):206–13. Epub 2002/07/20. doi: 10.1006/cyto.2002.1034. 12126643

[22] Zhang D, Sun M, Samols D, Kushner I. STAT3 participates in transcriptional activation of the C-reactive protein gene by interleukin-6. J Biol Chem. 1996;271(16):9503–9. Epub 1996/04/19. doi: 10.1074/jbc.271.16.9503. 8621622

[23] Amlani A, Barber C, Fifi-Mah A, Monzon J. Successful Treatment of Cytokine Release Syndrome with IL-6 Blockade in a Patient Transitioning from Immune-Checkpoint to MEK/BRAF Inhibition: A Case Report and Review of Literature. Oncologist. 2020;25(7):e1120–e3. Epub 2020/04/28. doi: 10.1634/theoncologist.2020-0194. 32337758 PMC7356700

[24] Yoshino K, Nakayama T, Ito A, Sato E, Kitano S. Severe colitis after PD-1 blockade with nivolumab in advanced melanoma patients: potential role of Th1-dominant immune response in immune-related adverse events: two case reports. BMC Cancer. 2019;19(1):1019. Epub 2019/10/31. doi: 10.1186/s12885-019-6138-7. 31664934 PMC6819390

[25] Schalper KA, Carleton M, Zhou M, Chen T, Feng Y, Huang SP, Walsh AM, Baxi V, Pandya D, Baradet T, Locke D, Wu Q, Reilly TP, Phillips P, Nagineni V, Gianino N, Gu J, Zhao H, Perez-Gracia JL, Sanmamed MF, Melero I. Elevated serum interleukin-8 is associated with enhanced intratumor neutrophils and reduced clinical benefit of immune-checkpoint inhibitors. Nat Med. 2020;26(5):688–92. Epub 2020/05/15. doi: 10.1038/s41591-020-0856-x. 32405062 PMC8127102

[26] Zhang Y, Chandra V, Riquelme Sanchez E, Dutta P, Quesada PR, Rakoski A, Zoltan M, Arora N, Baydogan S, Horne W, Burks J, Xu H, Hussain P, Wang H, Gupta S, Maitra A, Bailey JM, Moghaddam SJ, Banerjee S, Sahin I, Bhattacharya P, McAllister F. Interleukin-17-induced neutrophil extracellular traps mediate resistance to checkpoint blockade in pancreatic cancer. J Exp Med. 2020;217(12). Epub 2020/08/30. doi: 10.1084/jem.20190354. PMC7953739 32860704

[27] Tarhini AA, Zahoor H, Lin Y, Malhotra U, Sander C, Butterfield LH, Kirkwood JM. Baseline circulating IL-17 predicts toxicity while TGF-beta1 and IL-10 are prognostic of relapse in ipilimumab neoadjuvant therapy of melanoma. J Immunother Cancer. 2015;3:39. Epub 2015/09/18. doi: 10.1186/s40425-015-0081-1. 26380086 PMC4570556

[28] Singh S, Xiao Z, Bavisi K, Roszik J, Melendez BD, Wang Z, Cantwell MJ, Davis RE, Lizee G, Hwu P, Neelapu SS, Overwijk WW, Singh M. IL-1alpha Mediates Innate and Acquired Resistance to Immunotherapy in Melanoma. J Immunol. 2021;206(8):1966–75. Epub 20210315. doi: 10.4049/jimmunol.2000523. 33722878 PMC8023145

[29] Jaillon S, Ponzetta A, Di Mitri D, Santoni A, Bonecchi R, Mantovani A. Neutrophil diversity and plasticity in tumour progression and therapy. Nat Rev Cancer. 2020;20(9):485–503. Epub 20200721. doi: 10.1038/s41568-020-0281-y. 32694624

[30] Kapanadze T, Medina-Echeverz J, Gamrekelashvili J, Weiss JM, Wiltrout RH, Kapoor V, Hawk N, Terabe M, Berzofsky JA, Manns MP, Wang E, Marincola FM, Korangy F, Greten TF. Tumor-induced CD11b(+) Gr-1(+) myeloid-derived suppressor cells exacerbate immune-mediated hepatitis in mice in a CD40-dependent manner. Eur J Immunol. 2015;45(4):1148–58. Epub 20150223. doi: 10.1002/eji.201445093. 25616156 PMC4425346

[31] Fan YC, Fong YC, Kuo CT, Li CW, Chen WY, Lin JD, Burtin F, Linnebacher M, Bui QT, Lee KD, Tsai YC. Tumor-derived interleukin-1 receptor antagonist exhibits immunosuppressive functions and promotes pancreatic cancer. Cell Biosci. 2023;13(1):147. Epub 20230810. doi: 10.1186/s13578-023-01090-8. 37563620 PMC10416534

[32] Das S, Shapiro B, Vucic EA, Vogt S, Bar-Sagi D. Tumor Cell-Derived IL1beta Promotes Desmoplasia and Immune Suppression in Pancreatic Cancer. Cancer Res. 2020;80(5):1088–101. Epub 20200108. doi: 10.1158/0008-5472.CAN-19-2080. 31915130 PMC7302116

[33] Takahashi R, Macchini M, Sunagawa M, Jiang Z, Tanaka T, Valenti G, Renz BW, White RA, Hayakawa Y, Westphalen CB, Tailor Y, Iuga AC, Gonda TA, Genkinger J, Olive KP, Wang TC. Interleukin-1beta-induced pancreatitis promotes pancreatic ductal adenocarcinoma via B lymphocyte-mediated immune suppression. Gut. 2021;70(2):330–41. Epub 20200510. doi: 10.1136/gutjnl-2019-319912. 32393543

[34] Aggen DH, Ager CR, Obradovic AZ, Chowdhury N, Ghasemzadeh A, Mao W, Chaimowitz MG, Lopez-Bujanda ZA, Spina CS, Hawley JE, Dallos MC, Zhang C, Wang V, Li H, Guo XV, Drake CG. Blocking IL1 Beta Promotes Tumor Regression and Remodeling of the Myeloid Compartment in a Renal Cell Carcinoma Model: Multidimensional Analyses. Clin Cancer Res. 2021;27(2):608–21. Epub 20201104. doi: 10.1158/1078-0432.CCR-20-1610. 33148676 PMC7980495

[35] Herremans KM, Szymkiewicz DD, Riner AN, Bohan RP, Tushoski GW, Davidson AM, Lou X, Leong MC, Dean BD, Gerber M, Underwood PW, Han S, Hughes SJ. The interleukin-1 axis and the tumor immune microenvironment in pancreatic ductal adenocarcinoma. Neoplasia. 2022;28:100789. Epub 20220405. doi: 10.1016/j.neo.2022.100789. 35395492 PMC8990176

[36] Dosch AR, Singh S, Dai X, Mehra S, Silva IC, Bianchi A, Srinivasan S, Gao Z, Ban Y, Chen X, Banerjee S, Nagathihalli NS, Datta J, Merchant NB. Targeting Tumor-Stromal IL6/STAT3 Signaling through IL1 Receptor Inhibition in Pancreatic Cancer. Mol Cancer Ther. 2021;20(11):2280–90. Epub 20210913. doi: 10.1158/1535-7163.MCT-21-0083. 34518296 PMC8571047

[37] Theivanthiran B, Evans KS, DeVito NC, Plebanek M, Sturdivant M, Wachsmuth LP, Salama AK, Kang Y, Hsu D, Balko JM, Johnson DB, Starr M, Nixon AB, Holtzhausen A, Hanks BA. A tumor-intrinsic PD-L1/NLRP3 inflammasome signaling pathway drives resistance to anti-PD-1 immunotherapy. J Clin Invest. 2020;130(5):2570–86. doi: 10.1172/JCI133055. 32017708 PMC7190922

[38] Barbier L, Ferhat M, Salame E, Robin A, Herbelin A, Gombert JM, Silvain C, Barbarin A. Interleukin-1 Family Cytokines: Keystones in Liver Inflammatory Diseases. Front Immunol. 2019;10:2014. Epub 2019/09/12. doi: 10.3389/fimmu.2019.02014. 31507607 PMC6718562

[39] Ager CR, Boda A, Rajapakshe K, Lea ST, Di Francesco ME, Jayaprakash P, Slay RB, Morrow B, Prasad R, Dean MA, Duffy CR, Coarfa C, Jones P, Curran MA. High potency STING agonists engage unique myeloid pathways to reverse pancreatic cancer immune privilege. J Immunother Cancer. 2021;9(8). Epub 2021/08/04. doi: 10.1136/jitc-2021-003246. PMC8330562 34341132

